# Supplementary material for: Plant-Based Burgers in the Spotlight: A Detailed Composition Evaluation and Comprehensive Discussion on Nutrient Adequacy
Source: Foods. 2025 Jan 23;14(3):372. doi: 10.3390/foods14030372 (PMC11817254; doi:10.3390/foods14030372)
Supplement: Supplementary file 1 [file foods-14-00372-s001.zip › foods-3386909-supplementary.pdf]

Supplementary Material

Plant-Based Burgers in the Spotlight: A Detailed Composition Evaluation and Comprehensive Discussion on Nutrient Adequacy

Katia Regina Biazotto [1,2], Ana Carolina Hadlich Xavier [3], Rosane Ribeiro de Mattos [4], Júnior Mendes Furlan [4], Roger Wagner [3], Daniel Henrique Bandoni [1] and Veridiana Vera de Rosso [1,*]

Table S1. Determined values for typical diet (NIST-SRM1548B) expressed in mean ± SD, n = 3, and calculated trueness (%).

Component (g/100 g)	Determined Value	(%) Trueness
**Moisture**	0.43 ± 0.02	95.5
**Ash**	3.46 ± 0.05	97.5
**Protein**	14.25 ± 0.51	95.6
**Total fat**	26.77 ± 0.14	101.0
**Fibers**	5.04 ± 0.06	105.0
**Mineral (mg/100 g)**		
**Iron**	3.28 ± 0.31	113.8
**Sodium**	609.53 ± 27.41	87.2
**Calcium**	160.40 ± 0.83	99.4
**Potassium**	595.00 ± 43.05	106.7
**Manganese**	0.63 ± 0.02	131.2
**Magnesium**	53.80 ± 4.10	96.1
**Zinc**	1.91 ± 0.11	68.0
**Copper**	0.20 ± 0.01	125.0
**Phosphorus**	241.0 ± 12.0	108.0
